# A data landscape: picturing the residential and environmental features of the places where children and young people live in Wales, UK.

**DOI:** 10.23889/ijpds.v9i5.2598

**Published:** 2024-09-18

**Authors:** Rowena Bailey, Jo Davies, Rebecca Pedrick-Case, Amy Mizen, Rhodri Johnson, Richard Fry, Lucy Griffiths

**Affiliations:** 1 Swansea University

## Background

Natural landscapes and built environments are integral to human health and well-being. Place based interventions which target modifiable risk factors, such as obesity, during childhood development can have positive and enduring effects on individual health outcomes, reducing the risk of non-communicable disease in later life, and providing lasting societal gains from improved levels of population health.

## Objective

Our objective was to describe and quantify geo-spatial variation of the environments where children and young people (CYP) (aged up to 18 years) live, with the aim of informing national policies.

## Approach

Using routinely collected and linked administrative data held within the SAIL Databank, we characterised places of residences for every individual child from birth. Complete address history was used to quantify residential mobility of children and young people, as well as the household composition they live within. In addition, we have linked geo-spatial data and tracked changes to environmental and social-economic characteristics of areas which children move between.

## Results

Overall, 58% of CYP moved at least once before turning 18, and 75% of all house moves were to areas with the same or higher levels of deprivation. Using sophisticated visualisation methods to produce graphical representations, we have shown how these complex and interacting factors vary across the population.

## Conclusion

Visual descriptions of complex data provide a population level perspective of the places where children and young people live. This approach can help to inform policies which aim to provide equitable access to healthy environments which enable children to thrive and grow.

